# Control of Inhibition-Stabilized Oscillations in Wilson-Cowan Networks with Homeostatic Plasticity

**DOI:** 10.3390/e27020215

**Published:** 2025-02-19

**Authors:** Camille Godin, Matthew R. Krause, Pedro G. Vieira, Christopher C. Pack, Jean-Philippe Thivierge

**Affiliations:** 1 School of Psychology, University of Ottawa, 156 Jean-Jacques Lussier, Ottawa, ON K1N 6N5, Canada; 2 Department of Neurology and Neurosurgery, Montreal Neurological Institute, McGill University, Montreal, QC H3A 2B4, Canada; 3 Brain and Mind Research Institute, University of Ottawa, 451 Smyth Rd., Ottawa, ON K1H 8M5, Canada

**Keywords:** neural oscillations, Wilson-Cowan, inhibitory-stabilized network, homeostatic plasticity, damped oscillations, asynchronous quenching

## Abstract

Interactions between excitatory and inhibitory neurons in the cerebral cortex give rise to different regimes of activity and modulate brain oscillations. A prominent regime in the cortex is the inhibition-stabilized network (ISN), defined by strong recurrent excitation balanced by inhibition. While theoretical models have captured the response of brain circuits in the ISN state, their connectivity is typically hard-wired, leaving unanswered how a network may self-organize to an ISN state and dynamically switch between ISN and non-ISN states to modulate oscillations. Here, we introduce a mean-rate model of coupled Wilson-Cowan equations, link ISN and non-ISN states to Kolmogorov-Sinai entropy, and demonstrate how homeostatic plasticity (HP) allows the network to express both states depending on its level of tonic activity. This mechanism enables the model to capture a broad range of experimental effects, including (i) a paradoxical decrease in inhibitory activity, (ii) a phase offset between excitation and inhibition, and (iii) damped gamma oscillations. Further, the model accounts for experimental work on asynchronous quenching, where an external input suppresses intrinsic oscillations. Together, findings show that oscillatory activity is modulated by the dynamical regime of the network under the control of HP, thus advancing a framework that bridges neural dynamics, entropy, oscillations, and synaptic plasticity.

## 1. Introduction

In the mammalian brain, regions including the hippocampus and neocortex are comprised of interacting populations of excitatory and inhibitory neurons connected in a reciprocal fashion [1]. In these circuits, the balance between excitation and inhibition is key to controlling their dynamical regime of activity, ranging from stable firing rates [2] to oscillations [3] and irregular fluctuations [4].

A key feature of brain circuits is their ability to modulate neural oscillations under different states of activity. In awake cortical activity, oscillations force inhibitory interneurons (I) to fire in-phase with excitatory (E) neurons [5]. However, under anesthesia and other brain states, E and I populations exhibit out-of-phase activity, such that the phase of E neurons precedes I neurons by several milliseconds [6,7]. This phase shift plays an important role in neural computation by regulating the flow of information across distinct populations [8,9,10].

Theoretical work has captured these results using inhibition-stabilized networks (ISN) [11]. These networks are defined by a strong recurrent excitation that is compensated by inhibition such that if the latter is removed, the network becomes dynamically unstable. ISNs exhibit oscillations where E and I populations fire out of phase. Conversely, if recurrent excitation is weakened, the resulting non-ISNs exhibit in-phase oscillations between E and I neurons [12,13]. Thus, alterations in the state of the network account for changes in oscillatory activity [14,15,16,17]. Further, ISNs have successfully captured the response of neural circuits to external stimulation [18,19] as well as damped gamma oscillations (30–50 Hz range) [20,21,22,23].

However, in ISNs, the strength of connections between E and I populations is typically hard-wired and fixed. This aspect of the model runs contrary to both experimental and theoretical evidence suggesting that cortical networks can self-organize to an ISN state through homeostatic plasticity (HP) [24]. According to HP, synaptic weights of E and I neurons are adjusted to reach a target level of activity reflecting both the intrinsic activation of the network and its response to peripheral stimuli [25].

In this work, we examined the contribution of HP to the control of oscillatory activity in ISNs. Because HP is dependent upon the tonic levels of activity in the network, we reasoned that altering the tonic activity of an ISN will alter its regime of activity [26]. Although theory suggests that modulating tonic activity can shift a network from a low to a high state of activity [12], its impact on oscillations remains unclear.

We devised a model with excitatory and inhibitory populations described by canonical Wilson-Cowan equations where synaptic connections are subjected to HP. The ability of the model to capture key effects observed in experiments was examined, including: (i) a paradoxical decrease in inhibitory activity following the activation of inhibitory neurons; (ii) an increase in the phase coupling of E and I neurons under inhibitory periodic forcing; and (iii) damped gamma oscillations. Going further, we employed ISNs to reproduce an effect termed asynchronous quenching (AQ), in line with recent experimental work on the interference between intrinsic oscillations and external inputs oscillating at similar or dissimilar frequencies [27]. Overall, the control of dynamical states by HP broadens our understanding of neuronal oscillations by showing how synaptic plasticity gives rise to unique regimes of brain activity.

## 2. Materials and Methods

**Figure 1:**
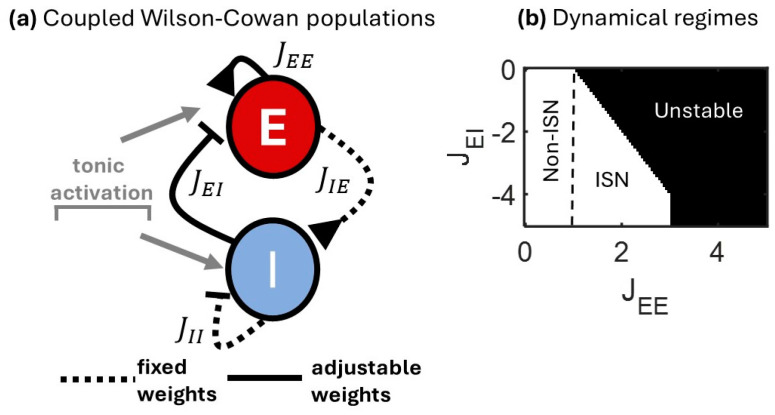
Mean-rate model of excitatory and inhibitory neurons exhibiting different dynamical regimes. (**a**) Wilson-Cowan circuit where a population of excitatory (E) neurons is coupled with inhibitory (I) neurons. Tonic activation is evenly applied to both populations. (**b**) The emergence of different dynamical regimes depends on $J_{EE}$ and $J_{EI}.$ Weak self-excitation ($J_{EE}$ < 1) results in a stable non-ISN regime, while stronger $J_{EE}$ yields either an ISN or unstable state.

The Wilson-Cowan formalism allows for a coarse-grained description of neural activity where a detailed characterization of individual neurons is replaced by the mean firing rate of large E and I populations [16]. Despite its apparent simplicity, the model can simulate rich dynamics across a variety of regimes [28]. Firing rates of the E and I populations (Figure 1a) are described by

(1) $$\tau \frac{dR_{E}}{dt}=-\alpha R_{E}+\phi \left(J_{EE}R_{E}+J_{EI}R_{I}+I_{ext}+I_{osc}^{E}+\sqrt{\tau }\xi^{2}\eta_{E}\right),\;\tau \frac{dR_{I}}{dt}=-\alpha R_{I}+\phi \left(J_{II}R_{I}+J_{IE}R_{E}+I_{ext}+I_{osc}^{I}+\sqrt{\tau }\xi^{2}\eta_{I}\right),$$

where $R_{E}$ and $R_{I}$ are the firing rates of E and I neurons, respectively. The function $\phi \left(\cdot \right)$ performs a linear rectification, $\phi \left(x\right)=x$ if x>0, and $\phi \left(x\right)=0$ otherwise. This rectification is intended for the model to be comparable to related work [24] and is employed as a simplification of non-linear functions employed elsewhere [29]. Coupling strengths $J_{XY}$ indicate weighted connections from node Y to node X. The leak parameter α = 0.5 controls the decay rate of activity back to baseline levels. The terms $\sqrt{\tau }\xi \eta_{E}$ and $\sqrt{\tau }\xi \eta_{I}$ represent independently drawn zero-mean Gaussian noise scaled by the integration time constant (τ = 10 ms) and variance ($\xi^{2}$ = 0.05). The tonic activation $I_{ext}$ is shared across E and I neurons and is constant over time. The terms $I_{osc}^{E}$ and $I_{osc}^{I}$ are external oscillations injected into the E and I neurons, respectively, and remain at zero unless specifically noted in numerical simulations.

The use of a linear rectification function captures properties of biological systems where, in some instances, the activity of neurons is approximated by a linear response over a certain range [30,31]. The use of saturating non-linearities becomes relevant when a system operates in an unstable regime to prevent firing rates from increasing without bounds. Within a stable regime, linear approximations of neural population dynamics are common and include the use of linear response theory [32] as well as linear dimensionality reduction techniques to describe network activity [33]. With the use of a linear rectification function, only states with a positive activation ($R_{E}$ > 0 and $R_{I}$ > 0) are considered, which aligns with neuronal systems where negative firing rates are implausible. The use of non-saturating input-output functions has been studied in various contexts, including supralinear networks [34]. While a neuron’s firing rate will ultimately saturate, cortical neurons exhibit unsaturated responses over a broad range of activity [35].

In this model, different regimes of activity can be obtained by altering the strength of excitatory and inhibitory connections feeding into the E population ($J_{EE}$ and $J_{EI}$, respectively), keeping other connections fixed. While evidence suggests that all synaptic connections within this canonical circuit are subject to plasticity, we restricted plasticity to $J_{EE}$ and $J_{EI}$ for two reasons. First, it provides a straightforward interpretation for the modulation of ISN and non-ISN regimes [36]. Indeed, by manipulating the values of $J_{EE}$ and $J_{EI}$, one can cover a variety of regimes spanning ISN, non-ISN, and unstable states (Figure 1b). Second, while plasticity may be possible at all synapses, this does not mean that it is continuously applied everywhere within biological networks. In fact, experimental evidence suggests the presence of pathway-specific plasticity, where only select connections are subject to alterations in connection strength while others remain fixed [37,38]. It is, therefore, appropriate to consider how pathway-specific plasticity influences network dynamics in a modeled circuit.

## 3. Results

### 3.1. Steady State Analysis

The behavior of the proposed model can be studied by finding the steady state of Equation (1) (i.e., $\frac{dR_{X}}{dt}=0$), focusing on the positive part of the linear rectifying function. Assuming stable connection weights and the absence of noise, the fixed points of the model are provided by

(2) $$R_{E}^{*}=\frac{J_{EI}R_{I}^{*}+I_{ext}}{\alpha -J_{EE}},\;R_{I}^{*}=\frac{J_{IE}R_{E}^{*}+I_{ext}}{\alpha -J_{II}}.$$

To examine synaptic weights leading to negative real eigenvalues, we considered the Jacobian matrix

(3) $$G^{\left(R\right)}=\left[\begin{array}{cc} \frac{\partial F_{E}}{\partial R_{E}} & \frac{\partial F_{E}}{\partial R_{I}}\\ \frac{\partial F_{I}}{\partial R_{E}} & \frac{\partial F_{I}}{\partial R_{I}} \end{array}\right],$$

where $F_{E}=\frac{dR_{E}}{dt}$ and $F_{I}=\frac{dR_{I}}{dt}$. The eigenvalues of $G^{\left(R\right)}$ are determined by

(4) $$\lambda^{\left(R\right)}=\frac{tr(G^{\left(R\right)})\pm \sqrt{{tr(G^{\left(R\right)})}^{2}-4det(G^{\left(R\right)})}}{2},$$

given $tr\left(G^{\left(R\right)}\right)=\left(J_{EE}-\alpha \right)+\left(J_{II}-\alpha \right)$ and

(5) $$\det\left(G^{\left(R\right)}\right)=\left(J_{EE}-\alpha \right)+\left(J_{II}-\alpha \right)-J_{EI}J_{IE}.$$

Negative real eigenvalues require that $tr\left(G^{\left(R\right)}\right)$ < 0 and $\det\left(G^{\left(R\right)}\right)$ > 0. Rewriting the determinant explicitly,

(6) $$\left(J_{EE}-\alpha \right)+\left(J_{II}-\alpha \right)>J_{EI}J_{IE}.$$

For small values of $J_{EE}-\alpha$, the above condition implies that $J_{EI}J_{IE}$ must also be proportionally small to maintain a positive determinant. A more explicit bound is therefore

(7) $$J_{EI}J_{IE}<\min\left(\left(J_{EE}-\alpha \right)\left(\alpha -J_{II}\right),\left(\alpha -J_{EE}\right)\left(\alpha -J_{II}\right)\right),$$

which accounts for interactions between $J_{EE}$, $J_{II}$, $J_{EI}$, and $J_{IE}$. Solutions exist for weights that respect the above stability condition and can be employed in numerical simulations for both ISNs ($J_{EE}$ = 1.5, $J_{EI}$ = −1.2, $J_{IE}$ = 0.5, $J_{II}$ = −0.05) and non-ISNs (same parameters, but lowering self-excitation to $J_{EE}$ = 0.5).

Different regimes of activity based on $J_{EI}$ and $J_{EE}$ are shown in Figure 1b. With weak excitation ($J_{EE}<1$), the model is in a non-ISN state where inhibition is not required to achieve dynamical stability. With intermediate values of excitation ($1<J_{EE}<3$), dynamical stability is possible but requires the presence of inhibition, thus forming an ISN regime. When excitation is too strong ($J_{EE}>3$), activity becomes unstable regardless of inhibition. Thus, a simplified Wilson-Cowan model with E and I populations and pathway-specific alterations in coupling strength exhibits a variety of dynamical regimes.

### 3.2. Relation Between ISN and Entropy

The ISN and non-ISN states can be interpreted as states of low and high entropy, respectively. To examine the relation between stable points of activity and entropy, the network is linearized around the steady-state solution. Small deviations (δ) from the steady-state values are defined as

(8) $$\delta R_{E}=R_{E}-R_{E}^{*},\;\delta R_{I}=R_{I}-R_{I}^{*}.$$

Next, the Wilson-Cowan equations are linearized using a first-order Taylor expansion,

(9) $$\tau \frac{d(\delta R_{E})}{dt}=-\alpha \delta R_{E}+\phi^{\prime }\left(J_{EE}R_{E}^{*}+J_{EI}R_{I}^{*}+I_{ext}\right)\delta R_{E}+\phi^{\prime }\left(J_{EI}R_{I}^{*}+I_{ext}\right)\delta R_{I},\;\tau \frac{d(\delta R_{I})}{dt}=-\alpha \delta R_{I}+\phi^{\prime }\left(J_{II}R_{I}^{*}+J_{IE}R_{E}^{*}+I_{ext}\right)\delta R_{I}+\phi^{\prime }\left(J_{IE}R_{E}^{*}+I_{ext}\right)\delta R_{E},$$

where $\phi^{\prime }(x)$ is the derivative of the activation function evaluated at the steady-state values. The resulting linear system can be written as

(10) $$\frac{d}{dt}\left(\begin{array}{c} \delta R_{E}\\ \delta R_{I} \end{array}\right)=\frac{1}{\tau }\left(\begin{array}{cc} -\alpha +\phi^{\prime }\left(J_{EE}R_{E}^{*}+J_{EI}R_{I}^{*}+I_{ext}\right) & \phi^{\prime }\left(J_{EI}R_{I}^{*}+I_{ext}\right)\\ \phi^{\prime }\left(J_{IE}R_{E}^{*}+I_{ext}\right) & -\alpha +\phi^{\prime }\left(J_{II}R_{I}^{*}+J_{IE}R_{E}^{*}+I_{ext}\right) \end{array}\right)\left(\begin{array}{c} \delta R_{E}\\ \delta R_{I} \end{array}\right).$$

The Jacobian of this linearized system is

(11) $$G^{\left(\delta R\right)}=\left(\begin{array}{cc} -\alpha +\phi^{\prime }\left(J_{EE}R_{E}^{*}+J_{EI}R_{I}^{*}+I_{ext}\right) & \phi^{\prime }\left(J_{EI}R_{I}^{*}+I_{ext}\right)\\ \phi^{\prime }\left(J_{IE}R_{E}^{*}+I_{ext}\right) & -\alpha +\phi^{\prime }\left(J_{II}R_{I}^{*}+J_{IE}R_{E}^{*}+I_{ext}\right) \end{array}\right).$$

The Kolmogorov-Sinai entropy of this system is linked to the Lyapunov exponents, which are approximated by eigenvalues of the Jacobian at the linear fixed points. Denoting the eigenvalues of $G^{\left(\delta R\right)}$ by $\lambda_{i}^{\left(\delta R\right)}$, the entropy S is related to the sum of these eigenvalues,

(12) $$S\approx \sum_{i}\lambda_{i}^{\left(\delta R\right)}.$$

In a stable system, where both eigenvalues have negative real parts, this simplifies to

(13) $$S\approx -tr\left(G^{\left(\delta R\right)}\right),$$

where $tr\left(G^{\left(\delta R\right)}\right)=\lambda_{1}^{\left(\delta R\right)}+\lambda_{2}^{\left(\delta R\right)}$. Eigenvalues of the Jacobian are obtained by solving

(14) $$\det\left(G^{\left(\delta R\right)}-\lambda I\right)=0,$$

where I is the identity matrix. The trace of the Jacobian is given by

(15) $$tr\left(G^{\left(\delta R\right)}\right)=\frac{\partial F_{E}}{\partial R_{E}}+\frac{\partial F_{I}}{\partial R_{I}},$$

where

(16) $$\frac{\partial F_{E}}{\partial R_{E}}=\frac{1}{\tau }\left(-\alpha +J_{EE}\phi^{\prime }\left(x_{E}\right)\right),\;\frac{\partial F_{I}}{\partial R_{I}}=\frac{1}{\tau }\left(-\alpha +J_{II}\phi^{\prime }\left(x_{I}\right)\right),$$

with $x_{E}=J_{EE}R_{E}^{*}+J_{EI}R_{I}^{*}+I_{ext}$ and $x_{I}=J_{II}R_{I}^{*}+J_{IE}R_{E}^{*}+I_{ext}$. Thus, the trace of the Jacobian can be expressed as

(17) $$tr\left(G^{\left(\delta R\right)}\right)=\frac{1}{\tau }\left[-2\alpha +J_{EE}\phi^{\prime }\left(x_{E}\right)+J_{II}\phi \prime (x_{I})\right].$$

Assuming that ϕ′(x) is nonzero for both neurons, $tr\left(G^{\left(\delta R\right)}\right)$ directly depends on $J_{EE}$ and as such, the entropy is approximately proportional to the negative trace of the Jacobian,

(18) $$S\approx \frac{1}{\tau }\left[2\alpha -J_{EE}\phi^{\prime }\left(x_{E}\right)-J_{II}\phi^{\prime }\left(x_{I}\right)\right].$$

Hence, entropy decreases when $J_{EE}$ increases (while remaining within a stable state), moving the system from a non-ISN to an ISN state. Intuitively, a low $J_{EE}$ results in random-like patterns of activity where neurons behave largely independently. Increasing $J_{EE}$ creates neuronal correlations that decrease the system’s entropy, thus showing a link between stable network states and entropy.

### 3.3. Homeostatic Plasticity

To examine the extent to which HP can modulate the state of a network towards or away from an ISN regime, we examined a classic HP formulation where the strength of E and I connections was updated as follows [24],

(19) $$\Delta J_{EE}=\eta R_{E}\left(E_{set}-R_{E}\right),\;\Delta J_{EI}=-\eta R_{I}\left(E_{set}-R_{E}\right),$$

where η= 0.01 is a fixed learning rate, and $E_{set}$ is a predetermined set point. This set point represents the target level of neural activity that the system attempts to maintain through synaptic adjustments.

To examine the behavior of the HP rule, we employed a separation of timescales. This analysis assumes that neural activity evolves much faster than synaptic weights. This is a reasonable assumption given that neural activity fluctuates on a millisecond timescale while HP evolves over the course of hours [39]. The difference in timescales between neural activity and synaptic weights is thus greater than six orders of magnitude. Therefore, we consider that once HP has reached a stable state, changes in neural activity do not immediately affect synaptic weights. In other words, we focus on the dynamics of neural activity while assuming a fixed strength of connections. Nonetheless, to acknowledge that both activity and weights evolve synergistically, this analysis is termed a quasi-steady state (QSS) approximation [24], such that the stable points of neural activity are referred to as “quasi-stable” and not as “stable” in the traditional sense. To avoid excessive wording, quasi-stable states are herein referred to as stable states.

An overview of the approach is as follows. First, following QSS, firing rates are assumed to reach an instantaneous steady state after weight modification. Hence, for a given set of weights, we calculated the steady state of neural activity (Equation (2)). Second, we found the steady state solution of the synaptic plasticity subsystem (Equation (19)) after substituting the steady state of neural activity. Finally, we performed a linear stability analysis of this subsystem. If both eigenvalues have negative real parts, then the system was classified as stable under the HP learning rule. These steps constitute a well-studied approach that has been validated in mean-rate models with HP rules [24].

Following QSS, the steady state of activity is substituted into Equation (19),

(20) $$\Delta J_{EE}=\eta R_{E}^{*}\left(E_{set}-R_{E}^{*}\right),\;\Delta J_{EI}=-\eta R_{I}^{*}\left(E_{set}-R_{E}^{*}\right).$$

Substituting the fixed points of neural activity into the HP learning rule,

(21) $$\Delta J_{EE}=\eta \left(\frac{J_{EI}R_{I}^{*}+I_{ext}}{J_{EE}-\alpha }\right)\left(E_{set}-\frac{J_{EI}R_{I}^{*}+I_{ext}}{J_{EE}-\alpha }\right),\;\Delta J_{EI}=-\eta \left(\frac{J_{IE}R_{I}^{*}+I_{ext}}{J_{II}-\alpha }\right)\left(E_{set}-\frac{J_{EI}R_{I}^{*}+I_{ext}}{J_{EE}-\alpha }\right).$$

Next, the fixed points of Equation (21) are obtained as

(22) $$J_{EE}^{*}=\alpha -\frac{J_{EI}R_{I}^{*}+I_{ext}}{E_{set}},\;J_{EI}^{*}=\frac{E_{set}\left(\alpha -J_{EE}\right)-I_{ext}}{R_{E}^{*}}.$$

We see from Equation (22) that the fixed points $J_{EE}^{*}$ and $J_{EI}^{*}$ depend on both the set point of the HP rule ($E_{set}$) and the tonic activation of the network ($I_{ext}$). To check for consistency, we can substitute the fixed points of firing rates $R_{E}^{*}$ (Equation (2)) into the fixed points of weight $J_{EE}^{*}$, which after simplification helds $J_{EE}^{*}=J_{EE}$. A similar exercise where we substitute $R_{I}^{*}$ into the solution for $J_{EI}^{*}$ yields $J_{EI}^{*}=J_{EI}$ when assuming a steady state where $R_{I}^{*}$ = $R_{I}$.

A further verification is to ensure that $J_{EE}^{*}$ and $J_{EI}^{*}$ do not generate unstable firing rates when substituted into $R_{E}^{*}$ and $R_{I}^{*}$. The stability of the eigenvalues (Equation (4)) requires that $tr\left(G^{\left(R\right)}\right)\;$< 0 and $\det\left(G^{\left(R\right)}\right)\;$> 0, as described earlier. These constraints can be verified numerically to ensure that solutions for $J_{EE}^{*}$ and $J_{EI}^{*}$ lead to stable firing rates.

The stability of the HP rule itself (Equation (19)) is determined by the eigenvalues of the Jacobian matrix,

(23) $$G^{\left(J\right)}=\left[\begin{array}{cc} \frac{\partial D_{E}}{\partial J_{EE}} & \frac{\partial D_{E}}{\partial J_{EI}}\\ \frac{\partial D_{I}}{\partial J_{EE}} & \frac{\partial D_{I}}{\partial J_{EI}} \end{array}\right],$$

where $D_{E}=\Delta J_{EE}$ and $D_{I}=\Delta J_{EI}$, with elements

(24) $$\frac{\partial D_{E}}{\partial J_{EE}}=\frac{\eta \left(J_{EI}R_{I}^{*}+I_{ext}\right)}{{\left(\alpha -J_{EE}\right)}^{2}}\left(2R_{E}^{*}-E_{set}\right),\;\frac{\partial D_{E}}{\partial J_{EI}}=\frac{\eta R_{I}^{*}}{\alpha -J_{EE}}\left(E_{set}-2R_{E}^{*}\right),\;\frac{\partial D_{I}}{\partial J_{EE}}=\eta \frac{{\left(R_{I}^{*}\right)}^{2}}{{\left(\alpha -J_{EE}\right)}^{2}},\;\frac{\partial D_{I}}{\partial J_{EI}}=\eta \frac{{\left(R_{I}^{*}\right)}^{2}}{\alpha -J_{EE}}.$$

Eigenvalues of $G^{\left(J\right)}$ are given by

(25) $$\lambda^{\left(J\right)}=\frac{-\eta \frac{R_{E}^{*}R_{I}^{*}}{{\left(\alpha -J_{EE}\right)}^{2}}\;\pm \sqrt{{\left(\eta \frac{R_{E}^{*}R_{I}^{*}}{{\left(\alpha -J_{EE}\right)}^{2}}\right)}^{2}-4\eta^{2}\frac{{\left(R_{E}^{*}\right)}^{3}R_{I}^{*}+{R_{E}^{*}\left(R_{I}^{*}\right)}^{3}}{{\left(\alpha -J_{EE}\right)}^{4}}}}{2}.$$

We define acceptable solutions of the Wilson-Cowan model with HP as regions of parameter space in the synaptic weights $J_{XX}$ where three conditions are met. First, to respect Dale’s law, we ensure that $J_{EE}\;$> 0 and $J_{EI}\;$< 0, thus restricting neurons to remain either excitatory or inhibitory, as this has key implications for signal processing in neural networks [18,40]. Second, the eigenvalues of activity must be negative (Equation (4)). Third, the eigenvalues of the synaptic weights must be negative (Equation (25)). With these three conditions, the resulting weights must follow Dale’s law, the activity of the model must be stable, and the weight values themselves must form stable points.

**Figure 2:**
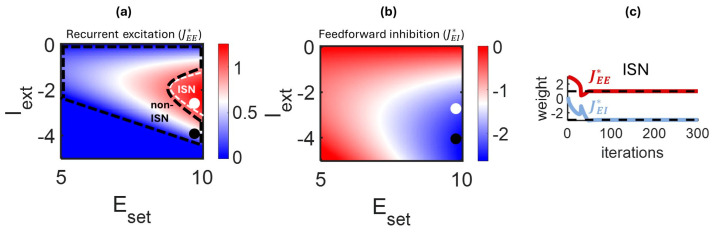
Homeostatic plasticity admits stable solutions for ISN and non-ISN regimes. The set point of HP ($E_{set}$) and tonic activation ($I_{ext}$) admit solutions (delineated by black and white dashed lines) that are stable and respect Dale’s law. Black and white circles provide an instance of each regime for recurrent excitation (**a**) and feedforward inhibition (**b**). For the parameters corresponding to the white circle in panel “a”, synaptic strengths settle to an ISN regime (**c**).

Fixed points of the HP rule ($J_{EE}^{*}$ and $J_{EI}^{*}$, Equation (22)) are shown in Figure 2a,b across a range of tonic activations ($I_{ext}$) and set points ($E_{set}$). The dashed lines in this figure delineate a region of parameter space where synaptic weights correspond to ISN and non-ISN regimes (Figure 1b). Within this region, we identified points (black and white circles) corresponding to instances of weights that fall within the ISN and non-ISN regimes. These points were found by taking the weight values for $J_{EE}^{*}$ and $J_{EI}^{*}$ and mapping them to Figure 1b, where these values were linked to the different dynamical regimes. A numerical example showing synaptic weights that gradually settle to a steady state ISN regime under the control of HP is shown in Figure 2c. Values of $E_{set}$ and $I_{ext}$ for this example are taken from Figure 2a,b (white circle). Within ~100 iterations, weights alter their initial values to settle into a stable state that matches the steady state solution (dashed lines).

Previous theoretical work has shown that a standard HP rule (Equation (19)) cannot yield a stable ISN regime [24]. However, this work did not consider the role of tonic activation ($I_{ext}$). If we consider the stable points $J_{EE}^{*}$ and $J_{EI}^{*}$ for a tonic activation of zero, we find that the solution does not respect Dale’s law and does not admit an ISN regime (Figure 2a,b, at $I_{ext}$ = 0). Hence, our model is consistent with previous results and further shows that non-zero tonic activation is required to attain an ISN state.

The ISN and non-ISN solutions identified in Figure 2a,b show that one can switch from these two regimes by keeping $E_{set}$ constant and simply altering tonic activation ($I_{ext}$). This is observed by the white dot (ISN) being stacked atop the black dot (non-ISN). In this way, a weak level of $I_{ext}$ gives rise to an ISN regime while a stronger negative value yields a non-ISN regime (note that it is also possible to obtain a non-ISN regime with $I_{ext}$ close to zero, but this state is not explored here). These results open the door to modulating the behavior of the network by controlling its tonic activation, an idea that will be exploited below to alter neural responses and oscillations.

### 3.4. Emergence of a Paradoxical Response

As a starting point, we tested whether the proposed model could capture a well-known feature of the ISN regime reported in experiments. When selectively increasing the drive of inhibitory neurons, ISNs exhibit a paradoxical *decrease* in their firing rate [11,19]. This effect has been captured by theoretical models where synaptic weights are hard-wired [13,36,40].

**Figure 3:**
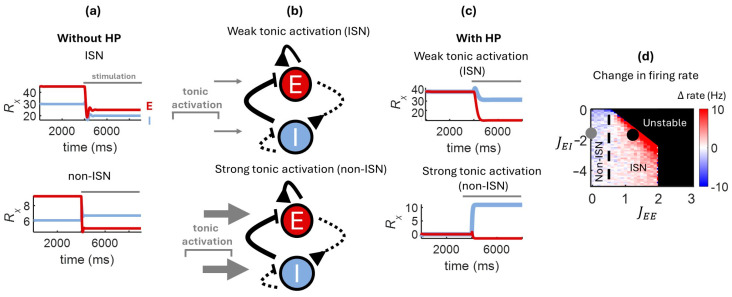
Paradoxical deactivation of inhibitory cells in the ISN regime. A model with no plasticity captures the well-known paradoxical response observed in ISNs (**a**). With HP, the strength of tonic activation determines the resulting coupling between E and I populations (**b**). While weak tonic activation results in an ISN regime exhibiting a paradoxical response, strong tonic activation yields a non-ISN regime with no such response (**c**). The change in firing rate (Δ rate) from baseline to stimulation shows combinations of excitatory and inhibitory couplings where the paradoxical response is strongest (**d**). Filled grey circle: instance of a non-ISN state; filled black circle: ISN state.

In a simplified version of the model where HP was omitted, this result was replicated by simply setting the weights to an ISN regime as mapped out in Figure 1b ($J_{EE}$ = 1.5, $J_{EI}$ = −2). In this scenario, external activation of the I population resulted in a decrease in inhibitory activity (Figure 3a). This effect occurs because stimulating the I population inhibits the E population, which in turn down-regulates the I population through feedforward excitation. Conversely, when weights were set to a non-ISN state ($J_{EE}$ = 0.5, $J_{EI}$ = −2), stimulating the I population yielded an increase in its activity. Thus, activating the inhibitory population yielded opposite effects in ISN and non-ISN states, recapitulating past experimental and theoretical findings [11,13,19,41,42,43,44,45,46].

To incorporate HP in the above simulations, we employed combinations of set point ($E_{set}$) and tonic activation ($I_{ext}$) that gave rise to an ISN or non-ISN regime. As mapped in Figure 2a,b, weaker tonic activation was employed to generate an ISN regime, and stronger tonic activation for a non-ISN regime (Figure 3b). The resulting values of $J_{EE}^{*}$ and $J_{EI}^{*}$ were then employed in numerical simulations of neural activity (Equation (1)).

With weak tonic activation, the network self-organized to an ISN state, yielding a paradoxical response to inhibitory stimulation (Figure 3c, top). Conversely, with a strong negative tonic activation, the network settled to a non-ISN state that did not exhibit a paradoxical response (Figure 3c, bottom). Thus, tonic activation was a determining factor in the regime of activity attained by HP. By modulating the strength of tonic activation, the network could occupy either an ISN regime characterized by a paradoxical response or a non-ISN regime where this response did not emerge.

To further examine the response of ISN and non-ISN regimes to inhibitory stimulation, we computed the change in firing rate (Δ rate) between the pre-stimulation steady state activity and the steady state obtained during stimulation. Positive values of Δ rates denote a *decrease* in the activity of the I population caused by stimulation, thus indicative of a paradoxical response. Positive values of Δ rate were concentrated near the border between ISN and unstable states, showing that paradoxical responses are characterized as an edge-of-stability effect [36] (Figure 3d). Formally, the change in inhibitory firing rate relative to an external input is termed the “gain” of inhibition,

(26) $$\frac{dR_{I}^{*}}{dI_{ext}}=\frac{\frac{1}{\alpha -J_{II}}\left(\frac{J_{IE}}{\alpha -J_{EE}}+1\right)I_{ext}}{1-\frac{J_{IE}J_{EI}}{\left(\alpha -J_{EE}\right)\left(\alpha -J_{II}\right)}},$$

and can be expressed as a Laurent series as $J_{II}$ goes to infinity,

(27) $$\frac{dR_{I}^{*}}{dI_{ext}}=\left(\left(\frac{1}{\alpha -J_{II}}\right)\frac{J_{IE}}{\alpha -J_{EE}}+1\right)\left(1+\frac{J_{IE}J_{EI}}{\left(\alpha -J_{EE}\right)\left(\alpha -J_{II}\right)}+{\left(\frac{J_{IE}J_{EI}}{\left(\alpha -J_{EE}\right)\left(\alpha -J_{II}\right)}\right)}^{2}+\ldots \right),$$

with a limit of

(28) $$\underset{J_{\mathrm{II}}\to \infty }{\lim}\frac{dR_{I}^{*}}{dI_{ext}}=0.$$

Thus, strong recurrent inhibition serves to stabilize firing rates in response to an external input. Intuitively, the implication of this result in terms of the model’s dynamics can be examined by re-writing the steady-state of inhibitory activity (Equation (2)) as

(29) $$R_{I}^{*}=\frac{\phi \left(J_{II}R_{I}^{*}+J_{IE}R_{E}^{*}+I_{ext}\right)}{\alpha },$$

by assuming that the input to the inhibitory neuron is passed through the linear rectification ϕ(·). In this case, as the self-inhibitory weight ($J_{II}$) increases, the rectification function becomes increasingly dominated by the self-feedback term $J_{II}R_{I}^{*}$, so that further changes in $I_{ext}$ have a negligible influence on the value of $R_{I}^{*}$.

In sum, an ISN regime near the edge of stability promotes the emergence of a paradoxical response where a stimulation applied to inhibitory neurons yields a decrease in their activity.

### 3.5. Phase Offset Induced by a Forced Oscillator

**Figure 4:**
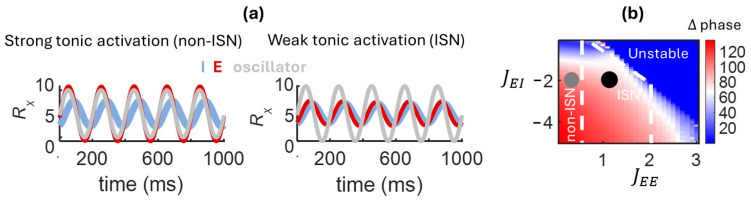
Phase offset between E and I populations in response to an external input. With a forced external oscillator, a tonic activation applied to I cells results in a large phase offset in a non-ISN state and a small offset in an ISN state (**a**). The phase offset (∆ phase) between E and I populations depends on the strength of excitatory and inhibitory couplings, which collectively determine the state of the network (**b**). Filled grey circle: non-ISN; filled black circle: ISN.

Next, we examined the response of the model to a periodic input by delivering a forced oscillation (5 Hz) to the I population [22]. With strong tonic activation, the network settled to a non-ISN state, as described earlier. In this state, the phase of the E population preceded the I population, resulting in out-of-phase activity (Figure 4a). By weakening tonic activation, however, the regime shifted to an ISN state where E and I populations fired in phase. These results capture and extend previous theoretical work where synaptic weights were hard-wired to an ISN or non-ISN regime [12,13]. In our model, HP allows weights to self-organize to a particular regime based on the level of tonic activation of the network. Thus, the model provides a simple, yet biologically plausible means of controlling the regime of the network, and hence the phase of synchronization between E and I. In the brain, alterations in tonic activation could be employed as a neuronal “switch” to gate signal propagation from external inputs to different regions of broadly distributed synaptic circuits.

A further distinction between ISN and non-ISN states relates to the phase between the forced oscillator and the two neural populations. In the non-ISN state, the E population was tightly coupled with the oscillator, while in the ISN state both populations were decoupled from the oscillator (Figure 4a). This result has implications for the ability of brain circuits to route information, given that tightly coupled activity promotes the propagation of inputs along neural pathways [8]. This effect was quantified by calculating the absolute phase difference between E and I populations,

(30) $$\Delta \;phase=\left|H\left(R_{E}\right)-H\left(R_{I}\right)\right|,$$

where H(·) is a Hilbert transform of neural activity, expressed in units of degree. The reduction in phase lag in the ISN state was more prominent near the border of instability, suggesting an edge-of-stability effect, though this effect was largely driven by changes in recurrent excitation ($J_{EE}$) (Figure 4b). As recurrent excitation increased and the network state shifted towards instability, the phase difference between E and I populations gradually decreased. In sum, numerical simulations captured and expanded past findings, showing how the oscillatory phase of E and I populations can be modulated by the network state under the control of synaptic plasticity.

### 3.6. Damped Oscillations

**Figure 5:**
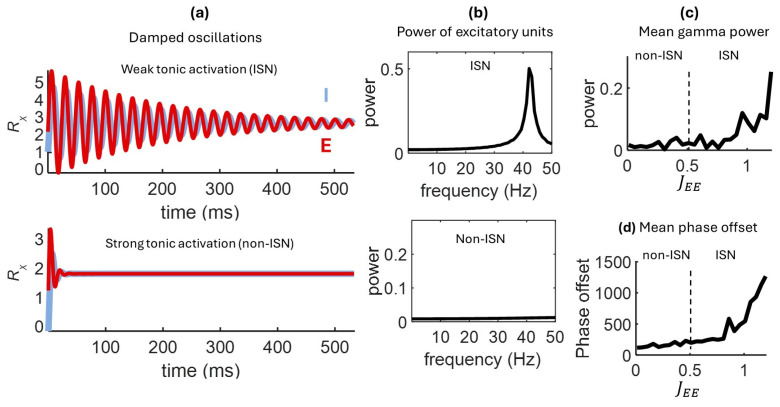
Damped oscillations in the ISN state. Damped oscillations are present in the ISN but not in the non-ISN regime (**a**), as shown by power spectra in both regimes (**b**). Mean gamma (30–50 Hz) power (**c**) and phase offset (**d**) increase with stronger excitatory coupling. In panels (**a**–**d**), damped oscillations were obtained by setting the decay rate of activity to α = 0.25. Weights were set to $J_{EE}$ = 0.5 (non-ISN) $J_{EE}$ = 1.5 (ISN), $J_{EI}$ = −1.5, $J_{IE}$ = 1.5, $J_{II}$ = −1.1.

The Wilson-Cowan model with HP exhibited damped gamma oscillations in the ISN state, where tonic activation was weak (Figure 5a,b). No oscillations were observed in the non-ISN state, where tonic activation was stronger. In these numerical simulations, gamma power (30–50 Hz) was modulated by the strength of recurrent excitation ($J_{EE}$), with higher gamma power obtained in an ISN state near instability (Figure 5c). This result provides further evidence for an edge-of-stability effect associated with the ISN regime, as shown in earlier results. The instantaneous phase offset between E and I populations increased as recurrent excitation was strengthened (Figure 5d).

Thus, damped gamma oscillations arose in the ISN state and increased in power near an unstable regime dominated by recurrent excitation. By modulating tonic activation, HP allowed the model to express either ISN or non-ISN regimes, providing a straightforward biological mechanism for the control of gamma oscillations.

### 3.7. Asynchronous Quenching

The presence of gamma oscillations in ISNs opens the possibility of studying AQ, referring to the interference between an intrinsic oscillation and an external input oscillating at a similar or different frequency. While the interference between oscillators has been studied in a broad range of fields, AQ specifically refers to the abolishing effect of an external force on an oscillation, which can be used to control various kinds of pathological or unwanted oscillations [47]. Importantly, AQ is applicable to self-exciting oscillators and manifests as a suppression of pre-existing oscillations. AQ is related to prior work on Arnold tongues that examined the effects of an external stimulation on neural oscillations [48,49,50]. A key distinction, however, is that AQ focuses on self-sustaining oscillations that occur in nonlinear oscillators, as opposed to weaker entrainment effects that are common in Arnold tongues.

**Figure 6:**
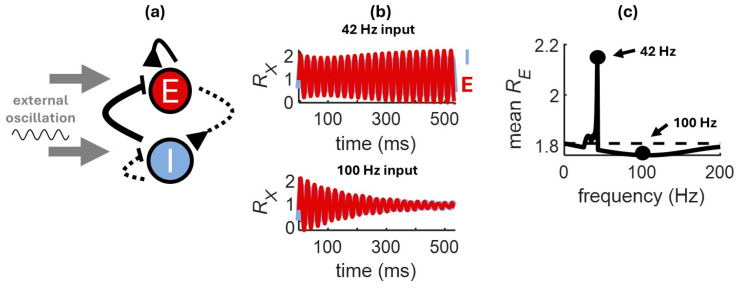
Asynchronous quenching of damped gamma oscillations. An external oscillator (amplitude: 0.045) was injected into both E and I cells of an ISN that produced damped oscillations (**a**). When the input matched the frequency of the damped oscillation, sustained activation was generated (top). A mismatched frequency yielded damped oscillations that decayed rapidly (bottom) (**b**). Summary of the effect of input frequency on the mean activity of E cells taken over a 500 ms window (**c**).

Here, we examined the effect of an external oscillation on the mean activity of the Wilson-Cowan model over time. Our starting point was an ISN that generated damped gamma oscillations (Figure 5a). A forced external oscillation was injected into both the E and I populations of this network (Figure 6a). When the frequency of the external signal closely matched the frequency of the damped gamma oscillation (42 Hz), the network generated sustained gamma waves (Figure 6b, top). However, when the external oscillation was higher in frequency, fluctuations decayed rapidly (Figure 6b, bottom). Hence, depending on the frequency of the external oscillation, the model exhibited either a higher or lower mean amplitude of activity (Figure 6c). Mean excitatory activity was highest when the intrinsic and extrinsic oscillations were closely matched in frequency, and lower when the intrinsic and extrinsic oscillations were detuned, reflecting a well-documented pattern of responses in cortex [27]. Importantly, an external oscillation that is slightly detuned from the intrinsic frequency can lead to a drastic reduction in mean amplitude, raising issues for experiments and clinical applications aimed at reducing aberrant brain oscillations.

## 4. Discussion

Theoretical models of neuronal circuits have examined how different regimes of activity emerge from the interaction between coupled excitatory and inhibitory populations. In these models, however, connectivity is usually hard-wired [13,36,41], unlike brain networks where synaptic plasticity modulates neuronal interactions [51]. Here, we incorporated HP in a Wilson-Cowan model with excitatory and inhibitory populations. Going beyond previous work [24], we showed how HP shapes properties of neural oscillations, including the relative phase of E and I populations and the power of gamma oscillations. Further, we proposed a simple biological mechanism whereby tonic activation can shift the regime of the network between ISN and non-ISN states. The model linked ISNs to entropy and replicated key experimental findings on paradoxical responses when driving inhibitory neurons, phase shifts under a forced oscillator, damped gamma oscillations, and asynchronous quenching. These findings were dependent on the state of the network and exhibited an edge-of-stability effect whereby they were strongly manifested near the border of instability.

At first glance, these results seem to contradict recent theoretical work showing that HP is unstable and hence cannot generate an ISN regime [24]. This past work, however, did not consider the role of tonic activation. Our findings show that the tonic activation of E and I populations is a determining factor in the solutions of the HP rule (Figure 2a,b), and that altering the set point alone ($E_{set}$) cannot bring the network to an ISN state. By adjusting the tonic activation and set point independently, one ends up with rules that are either stable or unstable, as well as rules that either respect or violate Dale’s law. Hence, our results help reconcile the contradiction between experimental findings that suggest that ISN is the default state of cortex [11] and theoretical work that suggests that standard HP rules are incompatible with an ISN regime.

A further distinction between our work and related efforts is that we restricted plasticity to two pathways—recurrent excitation and feedforward inhibition—while past models have applied it to all connections. While all synapses may be subject to plasticity, this does not imply that plasticity is applied continuously to all connections within biological networks, which would be energetically costly. Rather, brain circuits exhibit pathway-specific plasticity whereby synaptic changes are restricted to a subset of connections [38]. Our results show that applying plasticity to selected pathways captures a broad range of experimental findings related to ISNs and non-ISNs while facilitating the mapping between synaptic weights and dynamical states (Figure 1b). An important biological implication of these findings is that transitioning from one state to another does not require reconfiguring the synapses of an entire network.

Another implication of our findings concerns the control of neuronal activity in various practical applications of brain stimulation [27]. Our theoretical results suggest that it may be possible to alter the dynamical state of a network by selectively modulating its level of tonic activation. By doing so, it may be possible to suppress pathological network oscillations, for instance, by shifting the network from its default ISN state to a non-ISN state by lowering its tonic activity (Figure 5a) or by presenting an oscillation that is detuned from the intrinsically generated gamma activity (Figure 6b). This prospect is consistent with work that identifies regions of parameter space within a model where neural activity is susceptible to external control [48,49,50].

Substantial data suggests that AQ occurs in living brains. The strongest empirical evidence comes from direct neural recordings made during transcranial alternating current stimulation (tACS). This method is often thought to entrain neural activity, particularly when the ongoing baseline activity is unstructured [52,53]. However, when neurons are participating in a neural oscillation, applying tACS at a mismatched frequency instead desynchronizes them, a hallmark of AQ [24]. Similar hints of AQ can be found in the paradoxical results of other experiments as well: subtle visual flicker can cause event-related desynchronization [54], 40 Hz tone pips suppress 30 Hz gamma oscillations in auditory cortex [55], and rhythmic somatosensory stimulation disrupts ongoing alpha and beta oscillations in the primary somatosensory cortex [56].

This offers a straightforward alternative to existing closed-loop systems that continually monitor brain activity and apply counter-phased stimulation to destructively interfere with it. Although the responsive approach has had some success in invasive neuromodulation, it requires a complex apparatus that can accurately record neural activity while performing real-time signal processing, artifact rejection, and stimulus synthesis. These operations are particularly challenging for non-invasive methods, as the control signals (e.g., electroencephalograms) are noisy, especially during real-world use. In contrast, AQ exploits the brain’s ongoing dynamics to suppress oscillations and can, therefore, rely on less frequent measures and weaker stimulation. Models such as the one described here can help identify the requisite parameters and develop AQ into a robust therapeutic intervention [57].

At the same time, AQ may be an underappreciated factor in the (ir)reproducibility of many brain stimulation experiments [57]. Most use the same stimulation parameters for all participants, but brain oscillations vary between individuals and even within an individual over the course of an experiment. These fixed parameters may entrain neural activity in some subjects (or trials) while engaging AQ in others, leading to conflicting results. Achieving consistent results, especially in studies of neurological and psychiatric conditions where brain oscillations vary dramatically, may require more precisely targeted neuromodulation. AQ is thus both a serious confound and an exciting future direction for neuromodulation.

While our work focused on the role of HP in modulating dynamical regimes of activity across populations of neurons, there are several additional roles of HP that have not been considered here, including synaptic scaling [25], regulating neural activity [39], and guiding the development of neural circuits [58]. Hence, HP is a versatile learning rule that serves a variety of functions within both developing and mature neuronal circuits. The contribution of our work is to shed light on one aspect of HP, namely the ability to shape dynamical regimes to control the behavior of neuronal circuits.

To be sure, other synaptic rules influence network states, including Hebbian and other forms of plasticity [59]. Here, we focused on HP as a proof-of-concept that recurrent networks can self-organize to different dynamical regimes through pathway-specific plasticity by controlling their level of tonic activation. This mechanism constitutes a powerful means by which biological networks may modulate their activity and propagate information across broadly distributed circuits of the brain.

## Data Availability

The data that support the findings of this study are available from the corresponding author upon reasonable request.
